# Electrical Properties of Thiol-ene-based Shape Memory Polymers Intended for Flexible Electronics

**DOI:** 10.3390/polym11050902

**Published:** 2019-05-17

**Authors:** Christopher L. Frewin, Melanie Ecker, Alexandra Joshi-Imre, Jonathan Kamgue, Jeanneane Waddell, Vindhya Reddy Danda, Allison M. Stiller, Walter E. Voit, Joseph J. Pancrazio

**Affiliations:** 1 Department of Bioengineering, The University of Texas at Dallas, Richardson, TX 75080, USA; cfrewin@neuronexus.com (C.L.F.); Jonathan.Kamgue@utdallas.edu (J.K.); jeanneane@me.com (J.W.); VindhyaReddy.Danda@utdallas.edu (V.R.D.); allison.stiller@utdallas.edu (A.M.S.); Joseph.Pancrazio@utdallas.edu (J.J.P.); 2 Center for Engineering Innovation, The University of Texas at Dallas, Richardson, TX 75080, USA; Alexandra.Joshi-Imre@utdallas.edu (A.J.-I.); walter.voit@utdallas.edu (W.E.V.)

**Keywords:** Polymer, Dielectric, Resistivity, Permittivity, Curing, Corona-Kelvin

## Abstract

Thiol-ene/acrylate-based shape memory polymers (SMPs) with tunable mechanical and thermomechanical properties are promising substrate materials for flexible electronics applications. These UV-curable polymer compositions can easily be polymerized onto pre-fabricated electronic components and can be molded into desired geometries to provide a shape-changing behavior or a tunable softness. Alternatively, SMPs may be prepared as a flat substrate, and electronic circuitry may be built directly on top by thin film processing technologies. Whichever way the final structure is produced, the operation of electronic circuits will be influenced by the electrical and mechanical properties of the underlying (and sometimes also encapsulating) SMP substrate. Here, we present electronic properties, such as permittivity and resistivity of a typical SMP composition that has a low glass transition temperature (between 40 and 60 °C dependent on the curing process) in different thermomechanical states of polymer. We fabricated parallel plate capacitors from a previously reported SMP composition (fully softening (FS)-SMP) using two different curing processes, and then we determined the electrical properties of relative permittivity and resistivity below and above the glass transition temperature. Our data shows that the curing process influenced the electrical permittivity, but not the electrical resistivity. Corona-Kelvin metrology evaluated the quality of the surface of FS-SMP spun on the wafer. Overall, FS-SMP demonstrates resistivity appropriate for use as an insulating material.

## 1. Introduction

Thermoset thiol-ene and thiol-ene/acrylate shape memory polymers (SMPs) have been demonstrated lately as attractive substrate materials in flexible electronics. That includes organic and inorganic thin film transistors and diodes [1,2,3,4,5,6] as well as multi-electrode arrays for biomedical applications. [7,8,9,10,11,12] Shape memory polymers (SMPs) have the ability to change shape and/or softness in response to external stimuli [13,14,15,16,17] and therefore enable multifunctional and responsive devices. The use of highly tunable thiol click chemistry allows for the design of substrate and packaging materials having the desired mechanical and thermomechanical properties for a particular application. This can be achieved by controlling the cross-link density and the glass transition temperature (*T*_g_) of the polymer [7,18].

The fabrication of electronic circuits, semiconductors, and diodes on flexible polymeric substrates has been enabled by low-temperature processing techniques [19,20]. Our group successfully demonstrated the fabrication, electrical stability, and lifetime of hafnium oxide (HfO_2_) dielectric [2] and indium–gallium–zinc-oxide (IGZO) [1,3] thin film transistors on top thiol-ene/acrylate-based flexible substrates with softening capabilities. Additionally, SMP materials can be used for deployable devices, or parts thereof, and for the molding of specific device’s components. With this, the polymer can not only serve as a substrate, but also as an encapsulant or packaging material for electronic circuitries.

One example where responsive SMP substrates may be used is bioelectronics devices. In this case, the polymers have been engineered to transition from a stiffness of 2 GPa to as low as ~20 MPa in vivo [10,18,21,22,23,24,25]. These particular polymers undergo plasticization upon immersion into aqueous environments, and with this, their glass transition temperature (*T*_g_) shifts towards lower temperatures [23,24,26,27]. The associated transition in Young’s modulus allows SMP-based devices to be rigid during handling and insertion in air but soften to a more compliant modulus soon after implantation if the right polymer composition was chosen. For implantable devices that form intimate contact with soft tissue, the resulting interface with these SMP materials effectively decreases mechanical mismatch at the device–tissue interface. In the case of neural recording probes, commonly used devices have mechanical mismatch due to composition with stiff materials, like metal and silicon. The mismatch poses a source of constant inflammation and has been proposed as a basis for probe failure under chronic conditions [28,29,30]. Therefore, interest in softening SMPs as an integral material component for implantable bioelectronics devices has grown [10,14,31,32,33,34]. If SMPs are to serve not only as substrate material but also as an electrical insulation between electronic components, it is imperative to characterize their electric behavior.

Flexible electronic devices mainly consist of conductive traces surrounded by an electrically insulating material. As we consider the move to device miniaturization, these component layers, especially the insulation, can become thinned to the point that electrical properties, like polarization, breakdown, and resistivity becomes an extremely important consideration in device performance. Additionally, these properties are directly linked to the electrical issues of leakage, cross-talk, and parasitic shunt capacitance [35,36,37]. The goal of the present work was to characterize the electrical permittivity and resistivity of a representative thiol-ene SMP candidate. We have selected an SMP we named fully softening (FS-SMP) in previous studies [23,38] because it has a low glass transition temperature (between 40 and 60 °C dependent on the curing process), which allowed us to easily examine material parameters in the glassy as well as in the rubbery state. Additionally, we have considered two different curing approaches for the FS-SMP materials, which can affect the mechanical properties of the material [18,23].

The relative permittivity was 5.26 ± 0.11 and 6.38 ± 0.11, below and above *T*_g_ respectively, determined for FS-SMP cured under 254 nm wavelength (λ) ultraviolet (UV) radiation for 2 h. Reducing the curing exposure time under 254 nm UV to 3 min, followed by 1 h at 365 nm UV, increased the relative permittivity to 5.87 ± 0.08 below, and 7.79 ± 0.09 above *T*_g_. Unlike relative permittivity, resistivity for FS-SMP was only modestly altered between glassy and rubbery states. Surface resistivity, irrespective of cure, was just above 10^14^ Ω/sq in the glassy state, which was reduced by 10% in the rubbery state. The volume resistivity was reduced by an additional order of magnitude from 10^14^ Ω·cm in the glassy state to 10^13^ Ω·cm in the rubbery state. Finally, preliminary work with corona-Kelvin metrology, a non-contact method employed in the semiconductor industry to detect defects in dielectrics, suggested that while FS-SMP-spun films are heterogeneous, there were also large areas of homogeneity which might be suitable for device fabrication. 

## 2. Materials and Methods

### 2.1. Materials and Fabrication

**Figure 1:**
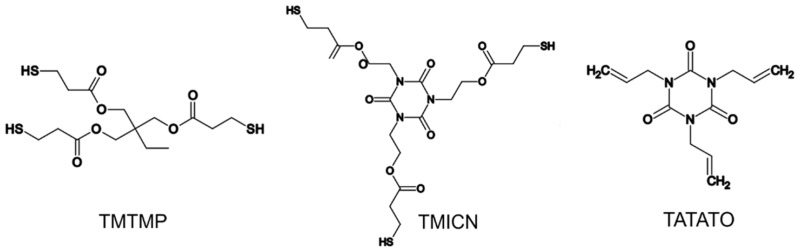
Chemical structures of monomers used for the synthesis of the fully softening shape memory polymer (FS-SMP).

The fabrication of FS-SMP was previously described [38]. The formulation described in Ecker et al. [23] was a slight variation thereof. The polymer is composed of a mixture of three monomer components (Figure 1): 0.5 mol% 1,3,5-Triallyl-1,3,5-triazine-2,4,6(1H,3H,5H)-trione (TATATO) (Sigma Aldrich, St. Louis, MO, USA), 0.45 mol% trimethylolpropane tris(3-mercaptopropionate) (TMTMP) (Sigma Aldrich, St. Louis, MO, USA), and 0.05 mol% Tris[2-(3-mercaptopropionyloxy)ethyl] isocyanurate (TMICN) (Evans Chemetics, Teaneck, NJ, USA). 2,2-dimethoxy-2-phenylacetophenone (DMPA) (Sigma Aldrich, St. Louis, MO, USA) was used as a photo-initiator. All of the chemicals were used as received without further purification.

FS-SMP was prepared by mixing exact mole fractions of the liquid monomers, TATATO, TMTMP, and TMICN, with 0.1 wt.% of DMPA into a vial at room temperature (~22 °C). The vial was covered in aluminum foil to prevent incident light from contacting the solution and mixed thoroughly using a DAC150.1 FV planetary speed mixer (FlackTec Inc., Landrum, SC, USA). The mixed solution was spin cast upon substrates consisting of a 100 mm diameter silicon wafer (University Wafers, Boston, MA, USA) using a Laurell WS-650-8B spin coater (Laurel Technologies, North Wales, PA, USA). Prior to spinning, each of the substrates had 20 nm of titanium followed by 400 nm of gold evaporated onto the surface using a Temescal 1800 single chamber evaporation system (FerroTec Inc, Santa Clara, CA, USA) to serve as the ground plate for the test capacitors.

**Figure 2:**
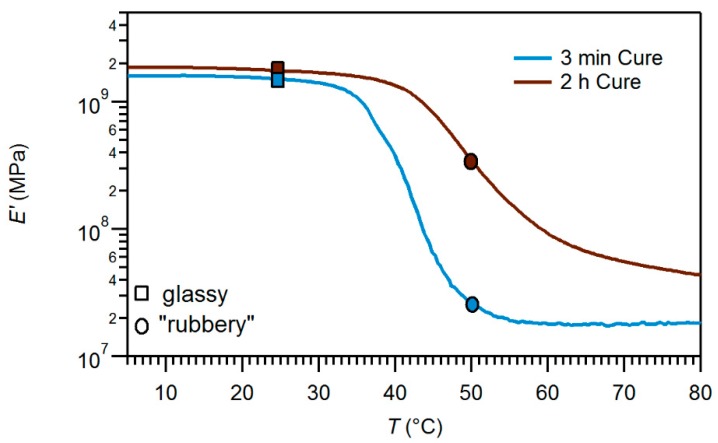
Graphs of storage modulus, *E*’, for fully softening shape memory polymers obtained using dynamic material analysis in air using the two curing methods discussed in this study. At temperatures below 37 °C, both curing formulations were in the glassy state, with *E*’ above 10^9^ Pa. However, while the 3 min curing process nearly reached its fully softened, rubbery state at 50 °C, the 2 h cure became only semi-soft with an *E*’ of ~500 MPa.

We have evaluated two curing methods for the FS-SMP polymer fabrication. Figure 2 demonstrates that the two processes produce equivalent moduli in the glassy and rubbery state but shifted *T*_g_. The first process, previously reported in [23], involved spinning the mixed monomers onto silicon substrates followed by exposure to 254 nm UV radiation at room temperature (~22 °C) and ambient atmosphere within an UVP CL-1000 cross-linking chamber (UVP LLC, Upland, CA, USA) containing five overhead UV bulbs for 120 min (2 h cure). The second polymerization condition was performed at ambient temperature under 254 nm with a reduced time of three minutes, which was immediately followed by 1 h of exposure to 365 nm UV light (3 min cure). At the completion of polymerization, the samples were placed in a vacuum oven, at 120 °C and 127.5 Torr, for 18 h to achieve complete curing. The resulting thickness of each wafer or slide was individually characterized to the nearest 0.1 µm using a Dektak 8 profilometer (Veeco, Plainview, NY, USA) to facilitate capacitance calculations.

**Figure 3:**
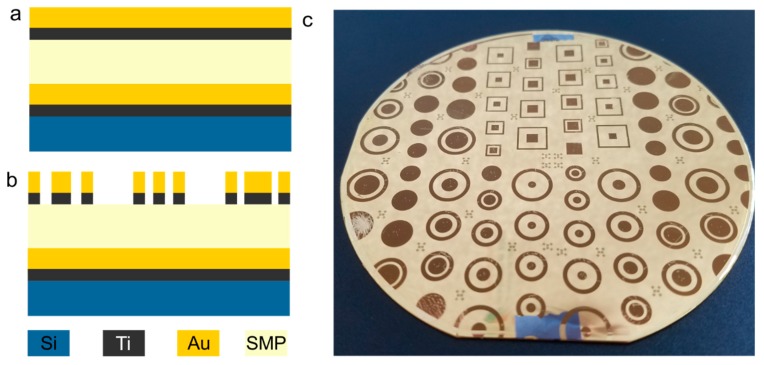
Fabrication of physical capacitors for testing. (**a**) shows a schematic of the sequence of the materials used for the fabrication of capacitors, (**b**) displays the stack after photolithography, and (**c**) shows a photograph of a typical device having differently sized capacitors.

To determine the electrical properties of FS-SMP, we crafted physical capacitors (Figure 3) for testing. The top plate of the physical capacitors and outer ring were fabricated using the following methodology. First, 20 nm of titanium, followed by 400 nm of gold, were blanket evaporated onto a silicon wafer. Next, FS-SMP was spin-coated and cured on top before another layer of Ti/Au was evaporated. The capacitors having sizes of 2, 3, and 4 mm, surrounded by 1 mm wide rings with gaps between of 0.5, 1, 1.5, and 2 mm, were patterned using photolithography via positive photoresist. The gold was wet-etched using Iodine-based etchant, and 10:1 Hydrofluoric acid solution was used to remove the underlying titanium adhesion layer.

### 2.2. Electrical Characterization

The electrical properties of resistivity and permittivity for SMP were measured and evaluated using guidelines published by the American Society for Testing and Materials (ASTM International) and the Association Connecting Electronics Industries (IPC) [39,40,41]. Round, parallel plate SMP capacitors were used for all measurements (Figure 3). The outer rings facilitated measurement of the leakage current across the surface of the material, while volume resistivity through the bulk of the SMP was measured between top and bottom plates. Leakage currents were used to calculate average surface and volume resistivity, while capacitance was used to calculate the relative permittivity for the FS-SMP [39,40,41]. In detail, capacitance was determined using the characteristic time (RC time constant *t*) required to charge a resistor-capacitor (RC) circuit. A carbon fiber resistor, characterized at 6,997 Ω, was connected in series with the top plate of the FS-SMP capacitor using a Model S-926 micro-positioner with a P pivot head and ST-T 5 µm point tungsten tips (Signatone, Gilroy, CA, USA). An RG-174/U coaxial cable containing a low-density polyethylene dielectric was utilized to connect the voltage source, a Keithley 3390 50 MHz Arbitrary Waveform Generator (Tektronix, Inc., Beaverton, OR, USA). Square wave pulses with an amplitude of 10 *V*_pp_ and periods of 0.01, 0.001, 1.0 × 10^−4^, and 1.0 × 10^−5^ s with equal high and low phases were continuously delivered. The output voltage waveforms were measured with a TBS 1052B Digital Oscilloscope Generator (Tektronix, Inc., Beaverton, OR, USA) using 10X attenuated voltage probes (TPP0051, Tektronix, Inc., Beaverton, OR, USA), which were characterized to have ~12 pF capacitance and 10 MΩ input resistance. The RC time constant was determined for the different square wave frequencies and the corresponding capacitance was calculated. These capacitances where then used to calculate the relative permittivity of the SMP-FS according to ASTM D150-11 [40], using the parallel plate equation.

Volumetric leakage current through the bulk of the material and surface current leakage through the top plate and outer ring electrode were measured using a 9103 USB Auto-Ranging Picoammeter (RBD Instruments, Bend, OR, USA) in response to DC voltage biasing. The 9103 provided a battery-powered voltage source of 90 V. The current was recorded every 50 ms for a total of 3 min using software provided by RBD Instruments. The initial current pulse was removed from the recordings, and the current was averaged across the total time period. The measured leakage currents were used to calculate the volume and surface resistivity according to ASTM 257-14 [39].

The electrical impedance of the wires and carbon resistors were considered in the calculations for all resistivity and permittivity calculations. Approximations for capacitance due to electrical fringing effects were removed using equations provided in the standards document [40].

Given that the FS-SMP was designed to operate over a specific temperature range, we examined the temperature dependence of the FS-SMP electrical properties. We placed the FS-SMP devices on a Fisherbrand^™^ Isotemp^™^ Stirring Hotplate, which included a Thermo Scientific FS PT100 external probe (Fisher Scientific, Waltham, MA, USA) for thermal feedback control. The polymer was heated to 50 °C, and the samples were held at this temperature for 10 min before measuring the capacitance and leakage currents again.

In total, two different wafers were fabricated, one for each polymer curing scenario, namely the 3 min and 2 h cure. Measurements were performed on individual, differently sized capacitors within these wafers. For the relative permittivity, *N* = 9 (2 h cure) and *N* = 14 (3 min cure) capacitors were measured at four different frequencies each. Thus, a total number of *N* = 36 (2 h cure) and *N* = 56 (3 min cure) measurements were performed at 22 and 50 °C, respectively. For the surface resistivity, *N* = 7 (2 h cure glassy), *N* = 8 (2 h cure rubbery), *N* = 18 (3 min cure glassy), and *N* = 16 (3 min cure glassy) measurements were performed. Volume resistivity was measured on *N* = 8 (2 h cure glassy), *N* = 8 (2 h cure rubbery), *N* = 19 (3 min cure glassy), and *N* = 16 (3 min cure rubbery) capacitors, respectively. All results are given as mean values ± standard deviation (SD).

### 2.3. Kelvin Force Probe Characterization

Non-contact voltage mapping has been used in the silicon circuit industry as a quick, non-destructive method to detect defects in insulators [42,43,44,45]. While the process has been described in detail previously, a brief explanation of the process is as follows. The Si wafer containing the FS-SMP thin film was placed on a grounded vacuum chuck. A 2 mm diameter vibrating Kelvin probe, accompanied with a vibrating reference electrode, was placed approximately 1 mm above the surface of the FS-SMP. The reference electrode is DC-biased and vibrates normally to the measured surface. The time-variation in capacitance, caused by the changes in the angular frequency of the vibration, was monitored using a lock-in amplifier in response to reference electric potential sweeps to determine the contact potential difference (*V*_cpd_). *V*_cpd_ was a series system composed of the potentials from the work function difference from the metal probe and references, the air insulation, the insulating film, and the semiconductor space charge region. The DC voltage applied to the reference electrode generated current due to the changes in the capacitance and was then adjusted to reduce the current to zero, at which point *V*_cpd_ = −*V*_DC_. Equipment calibration removed the contact potential of the Kelvin probe and reference electrode. Contributions from the potential due to the semiconductors surface barrier, *V*_SB_, were removed by subtracting the potential obtained under wafer illumination, producing excess carriers to reduce surface band bending, and the wafer recorded in the dark. The remaining potential was the contribution of the dielectric material, *V*_D_. As this potential is proportional to the dielectric charge-to-permittivity ratio, variations indicated changes in material electronic properties. The entire wafer surface was scanned by moving the wafer under the probe.

## 3. Results

**Figure 4:**
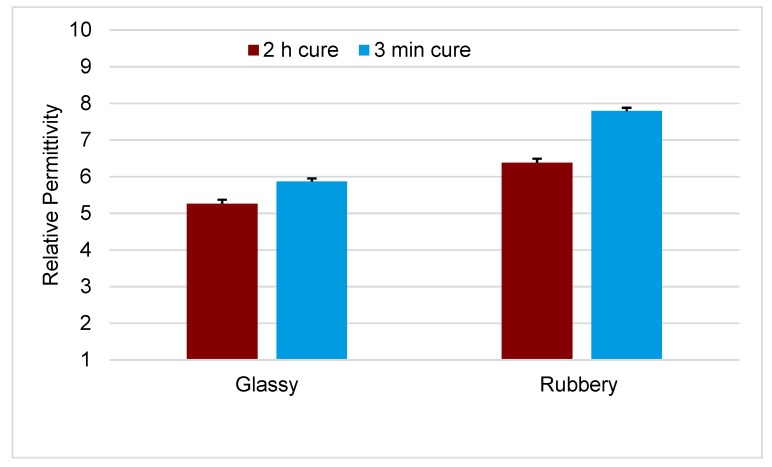
Mean relative permittivity of fully-softening shape memory polymer (FS-SMP) obtained at glassy and rubbery thermomechanical states. *N* = 36 (*N* = 9 capacitors with 4 replicate measurements each) for the 2 h cure FS-SMP and *N* = 56 (*N* = 14 capacitors with 4 replicate measurements each) for the 3 min cure FS-SMP. Standard deviation is represented through the positive error bars. The temperatures of measurement for the glassy state were 22 °C and for the rubbery, 50 °C.

Figure 4 displays the relative permittivity obtained for the two curing processes of FS-SMP evaluated at two temperatures that provide for the thermomechanical state of the FS-SMP. At room temperature, FS-SMP was in a glassy state, and the relative permittivity of the 2 h cure FS-SMP was 5.26 ± 0.11. The 3 min cure FS-SMP at the same temperature produced a permittivity of 5.87 ± 0.08. Increasing the temperature to change the state of the FS-SMP to rubbery demonstrated that the 2 h cure FS-SMP relative permittivity elevated to 6.38 ± 0.11, whereas the 3 min cure FS-SMP relative permittivity levels were 7.79 ± 0.09, respectively.

A three-way ANOVA was performed with a threshold of 0.05 between the variables of square-wave voltage signal frequency, the thermomechanical state of the polymer, and the curing method. Statistics for the tested factors are reported listing the *F* value, including the associated degree of freedom and *N* value separated in parenthesis, as well as the *p* value for the test. The permittivity was found to be statistically independent from changes in the frequency range from 100 to 10,000 Hz (*F*(3,332) = 0.02465, *p* = 0.99476). Thus, the measurements at the different frequencies were grouped together (Figure 4). Additionally, the interaction between frequency and thermomechanical state (*F*(3,332) = 0.62841, *p* = 0.5971), and the interaction for the factors for frequency, curing, and thermomechanical state (*F*(9,332) = 0.26987, *p* = 0.98231) demonstrated no statistically significant effect on the overall mean relative permittivity. Removing the frequency as an independent variable, we compared the factors of polymeric state (*F*(1,214) = 81.76, *p* = 9.4 × 10^−17^), and curing (*F*(3,214) = 92.61, *p* = 1.89 × 10^−38^), as well as the interactions between these factors (*F*(3,214) = 6.59, *p* = 2.81 × 10^−4^), and demonstrated evidence of significantly different mean values for relative permittivity.

In summary, the method of polymer curing, along with the thermomechanical state of the polymer, has an influence on the relative permittivity.

A post hoc Tukey test was used to investigate specific factors which influenced the relative permittivity at a threshold level of *p* < 0.05. The 3 min cure produced a significantly higher mean relative permittivity than the 2 h cure when both samples were evaluated within the same environment and the thermomechanical state remained constant. A change in thermomechanical state from glassy to rubbery produced a significant increase in relative permittivity. To summarize, the change in state from glassy to rubbery produced a significant increase in relative permittivity, the longer curing process significantly reduced molecular polarization within the FS-SMP.

**Figure 5:**
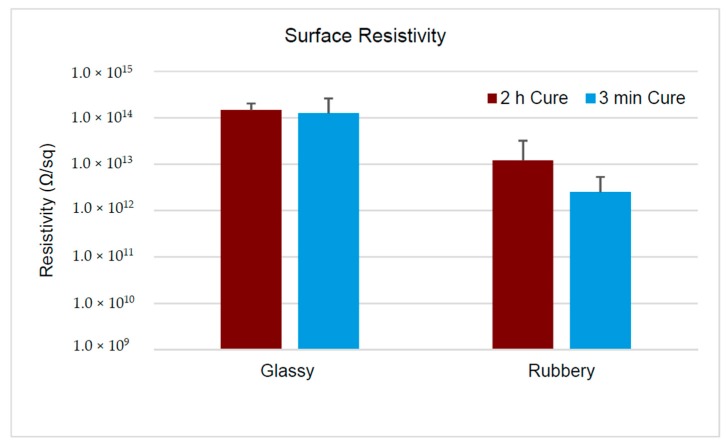

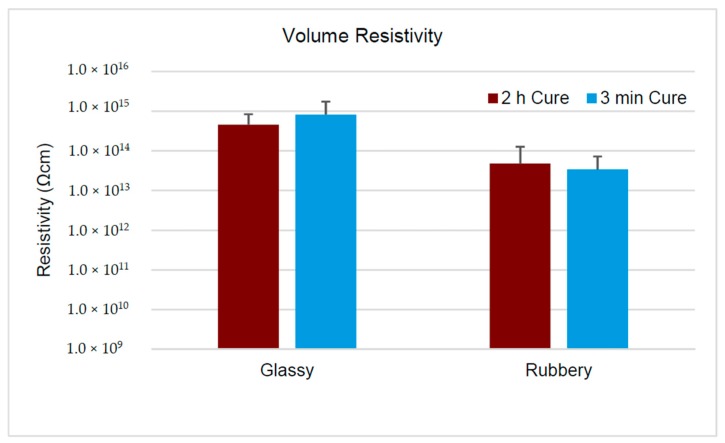
Two bar graphics displaying the mean surface and volume resistivity of FS-SMP, respectively. *N* = 7–8 (2 h cure) and *N* = 16–19 (3 min cure) measurements were performed for surface and volume resistivity at 22 and 50 °C, respectively. Standard deviation is represented through the positive error bars.

While the electric property of relative permittivity is an important factor in the consideration of AC leakages, electrical resistivity is an extremely important factor when considering DC leakages in an insulating material. We measured not only the resistivity of bulk FS-SMP, but also the resistivity across the skin, or surface, of the polymer. The mean surface and volume resistivity ± standard deviation of FS-SMP samples fabricated with the two curing techniques is displayed in Figure 5. The surface resistivity for the 2 h cure FS-SMP was 1.47 × 10^14^ ± 5.60 × 10^13^ Ω/sq in its glassy state, and 1.20 × 10^13^ ± 2.01 × 10^13^ Ω/sq in the rubbery state. The 3 min cure FS-SMP possessed a mean surface resistivity of 1.25 × 10^14^ ± 1.35 × 10^14^ Ω/sq below *T*_g_, and 2.50 × 10^12^ ± 2.72 × 10^12^ Ω/sq above *T*_g_. 

The volume resistivity in the glassy state was 4.55 × 10^14^ ± 3.83 × 10^14^ Ω·cm for the 2 h cure FS-SMP, and 8.13 × 10^14^ ± 9.24 × 10^14^ Ω·cm for the 3 min cure. The volume resistivity in the rubbery state was 4.75 × 10^13^ ± 7.99 × 10^13^ Ω·cm for the 2 h cure, and 3.37 × 10^13^ ± 3.87 × 10^13^ Ω·cm for the 3 min cure.

**Figure 6:**
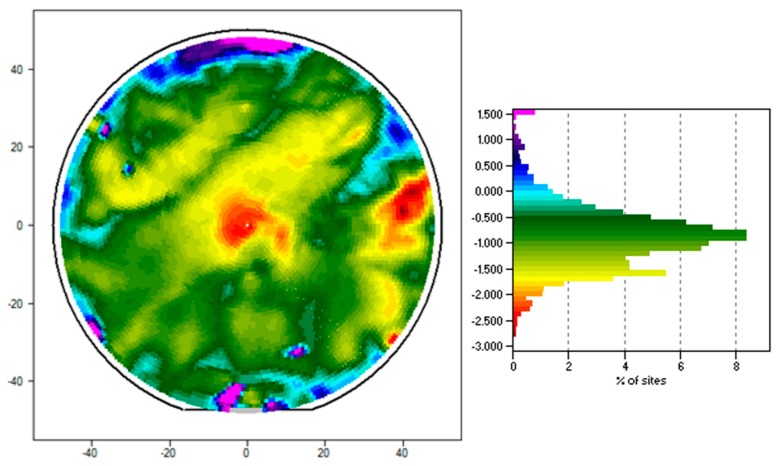
A contact potential voltage map (*V*_CPD_) displaying charge variation in a dielectric film of FS-SMP spun onto a 100 mm diameter p-doped silicon wafer and cured using the 3 min FS-SMP process.

Figure 6 displays an example of the contact potential voltage, *V*_CPD_, mapped across the surface of a 100 mm diameter, boron-doped P-type silicon wafer of 1 Ω·m resistivity, 7 µm thick film of FS-SMP, and cured with the 3 min FS-SMP methodology. *V*_CPD_ value ranges between 1.6 V and −2.8 V with an average of −0.885 V and a standard deviation of 0.691 V across the wafer. Positive *V*_CPD_ are mostly confined near the edges of the wafer. *V*_CPD_ below −2 V appear as islands, and the two dominant such islands are approximately 10–15 mm in size. There are large areas of *V*_CPD_ values possessing homogeneous potential.

## 4. Discussion

Relative permittivity in amorphous polymers has been theorized to arise from two main molecular interactions. The first is from charge polarization due to dipoles within the polymer chain. Another is the relaxation process which has been associated with the mobility of molecules and motion within portions of the main chain, side chains, or side groups [46]. This relaxation has been generally linked to thermal processes which rise in effective strength with an increase in temperature. As FS-SMP does not possess side chains or groups, one must consider the overall chain rigidity and the chemical composition of the polymer. It has been shown that these latter factors are extremely important in the formation of permanent dipoles and increase in relative permittivity [46,47].

Our study focuses on a single polymer chemistry under two different polymerization methods. As discussed previously, the method of polymerization by exposure to 254 nm wavelength UV light for two hours has demonstrated a wider transition window in FS-SMP as well as an up-shift of *T*_g_ by about 10 °C as compared to the 3 min method, which is attributed to differences in the cross-linking mechanism [18,23]. In the case of the 3 min method, where exposure to 254 nm wavelength UV radiation is limited to 3 min and polymerization is completed by subsequent exposure to 365 nm wavelength UV light, our DMA findings suggest a reduction in cross-link density and a more homogenous network formation (Figure 2). Although dielectric relaxation has been demonstrated to greatly increase at the glass transition temperature [47], we have seen a much larger proportional increase in relative permittivity during the physical transformation from glassy to rubbery for the 3 min cure process than the 2 h cure. Most likely, this effect is attributable to the lower overall curing dose (short amount of exposure to the high energy/lower wavelength followed by one hour of exposure to the lower energy/higher wavelength), as opposed to two hours exposure with the high energy/lower wavelength, which would create a polymer inherent with additional relaxation during cross-linking (less curing stress) and increased chain mobility compared to the 2 h cure. However, it should be noted that the 2 h cure is still within its transition temperature and not fully rubbery at the high-temperature measurement (50 °C). This might also contribute to the mobility of the polymer and associated relaxation. The hydrophobicity of both polymer films was similar. The contact angle of water droplets was measured to be (70.7 ± 5.5°) for the 2 h cure and (80.3 ± 2.9°) for the 3 min cure. Thus, the samples of the 3 min cure resulted in slightly higher hydrophobicity.

We found that the surface and volume resistivity of FS-SMP exceeded the thresholds of 1.0 × 10^12^ Ω/sq, and 1.0 × 10^9^ Ω·cm respectively, while in glassy or rubbery states and regardless of the curing methodology, and therefore classifies as an insulating polymer [48,49]. Since the mean and standard deviation values of measured resistivities were close together, we also looked at the sample percentiles (Table 1). We found that the majority (at least 90%) of the measured samples were above that threshold that classifies them as insulating polymer. The only exception was the 3 min cure FS-SMP in the rubbery state that had only 71% of the measured samples above the threshold of 1.0 × 10^12^ Ω/sq for the surface resistivity, while the volume resistivity was still above 90%.

When we compare the SMP-FS with other insulating materials at room temperature (Table 2), we can see that it falls well within their values for volume and surface resistivity.

Uniformity is an important aspect of processing micro- and nano-sized devices, and insulating materials should perform as designed. Kelvin force probe characterization is widely used in the semiconductor industry to quickly scan entire production wafers with the aims to ensure quality of dielectric materials, detect possible device destroying defects, and increase the product yield. We utilized this technique to assess charge distribution in spin-coated FS-SMP. The film was found to be mostly homogeneous at around −900 mV contact potential with some isolated area showing potential differences. Unfortunately, the measurement from the Kelvin probe process only allows detection of differences in potential. It is not possible to accurately use the method to attribute the changes to variations in surface thickness or to chemical defects within the matrix. Future investigations will be needed to characterize the mechanisms behind the potential variations. We also conducted atomic force microscopy (AFM) measurements to investigate the surface roughness of the samples and found that the spin coating resulted in relatively smooth polymer surfaces. The surface roughness of SMP samples after both curing scenarios was similar. AFM measurements revealed root mean square averages of height deviations of the assessed profile *R*_q_ of 0.291 and 0.316 nm for the 2 h and 3 min cure samples, respectively.

## 5. Conclusions

Thiol-ene and thiol-ene/acrylate-based shape memory polymers have been developed by our group to demonstrate desirable mechanical properties for flexible electronics, such as organic and inorganic thin film transistors and diodes, and multi-electrode arrays for biomedical applications. Although the mechanical properties have been reported across multiple investigations, the electrical properties have not been reported for this promising material.

Our investigations demonstrated that reducing the exposure of the FS-SMP monomer mixture to 254 nm UV light from 2 h to 3 min during polymerization changes the polymer network slightly and increases the relative permittivity: 5.3 to 5.8 in the glassy state. Relative permittivity measured in the rubbery state was consistently higher than that in the glassy state for both curing methods.

FS-SMP, according to the standards established by the ESD Association, displays good electrical properties when compared to other thin film materials. The surface and volume resistivity of the material classifies as an insulating polymer, independent of curing or thermomechanical state. FS-SMP may also be considered as a dielectric for use in flexible capacitors.

## Figures and Tables

**Table 1:** Mean values ± standard deviation (SD) and values for surface and volume resistivity at the 10th percentile.

Material	Surface Resistivity (Ω/sq)		Volume Resistivity (Ω·cm)	
	Mean ± SD	10th Percentile	Mean ± SD	10th Percentile
2 h cure glassy	1.47 × 10^14^ ± 5.60 × 10^13^	7.58 × 10^13^	4.55 × 10^14^ ± 3.83 × 10^14^	2.03 × 10^12^
2 h cure rubbery	1.20 × 10^13^ ± 2.01 × 10^13^	2.41 × 10^13^	4.75 × 10^13^ ± 7.99 × 10^13^	1.09 × 10^11^
3 min cure glassy	1.25 × 10^14^ ± 1.35 × 10^14^	1.23 × 10^13^	8.13 × 10^14^ ± 9.24 × 10^14^	1.33 × 10^12^
3 min cure rubbery	2.50 × 10^12^ ± 2.72 × 10^12^	2.44 × 10^11^	3.37 × 10^13^ ± 3.87 × 10^13^	3.22 × 10^9^

**Table 2:** Comparison of dielectric properties of various insulating materials from [50] if not stated differently.

Material	Volume Resistivity (Ω·cm)	Surface Resistivity (Ω/sq)
SMP-FS	10^14^	10^14^
Ceramics	10^11^–10^14^	-
Soda-lime glass	10^11^–10^13^	10^10^–10^12^
Hard rubber	10^15^–10^17^	10^10^–10^18^
Epoxy cast resin	10^14^–10^15^	10^7^ –>10^14^
Acrylic	>10^15^	>10^14^
Polypropylene	10^15^–10^17^	>10^15^
Parylene C	10^12^–10^16^ [51]	10^15^
Polyimide (Kapton)	10^17^ [52]	
